# Proceedings Richmond Academy of Medicine and Surgery

**Published:** 1895-11

**Authors:** Mark W. Parker

**Affiliations:** Secretary and Reporter; Richmond, Va.


### Society Notes.

### Proceedings Richmond Academy of Medicine and Surgery.

RICHMOND, VA., August 13, 1895.

Dr. V. W. Harrison, 1st Vice-President, in the chair.

Dr. Landon B. Edwards read a paper on the Diagnosis, Special and Differential, of Cholelithiasis. Stone may sometimes be formed in the liver itself,. The position of the gall-bladder varies with that of the liver. It can be recognized by palpation. Choletithiasis is rare in children under twelve, becoming more frequent as age advances, and being more common over thirty, and in women. It is comparatively frequent in the insane.

The characteristic symptoms suggesting gall stone are, multipara seized with a grinding pain radiating over the whole body. There are perspiration, retching, vomiting, dyspnœa, pulse lessened in volume, expression of pain, no rise in temperature. The symptoms do not intermit, and pain is not relieved unless the stone passes. It lasts two or three days, and leaves soreness. Ease is brought about through a pressure paralysis. Jaundice is not always present, but may occur gradually from occlusion. Mahogany urine may be present. The flow of bile being prevented, the skin becomes darker, vomiting ceases to be bilious, stools becomes pasty, and distension of the gall-bladder by mumus, may occur, simulating floating or displaced kidney.

Cholelithiasis is diagnosed from *malaria* by the mild fever, scarcely jaundiced hue, chills, etc.

From *catheterization*: The chills may become rigors, the fever rises, pulse slow—40 to 50, irregular, and not in keeping with the fever.

*Tumors, especially malignant pancreatic, and pancreatic stone:* Hemorrhages from the stomach, bowels, etc.; obstruction of the common duct, damming of the fluid in the gall-bladder and ducts, producing jaundice. Indigestion of fat is present in pancreatic troubles.

*Empyemia of the gall-bladder:* By the history of chills, fever, etc., with liver trouble.

*Adhesions of the gall-bladder* may so form as to cause discharge of bile into the duodenum direct, and there may be no suspicion of stone.

Symptoms of typhoid and other fevers, and of malignant and benignant tumors, are simulated by stone. It is said that when the stone passes from the cystic to the common duct, pain is lessened, and as soon as the passage occurs, mucus begins to be dammed into the body of the liver, causing its enlargement. This is diagnosed from *hepatitis* by less pain in the latter.

Heredity may lead to differentiation of stone. It is seldom that repetitions of hepatic colic do not occur.

Operations for removal should not be performed unless the typical symptoms are present.

Cases occur more frequently than is supposed, giving rise to symptoms elsewhere.

There is no medical treatment efficient for the disentegration or removal of gall-stone. The indication is to relieve pain, and for this, the best agent is, by far, hyoscyamine.

### DISCUSSION.

Dr. Hugh M. Taylor quoted Robson, who lays stress on the characteristic suddenness of the paroxysmal. In some instances, pain is referred to the left shoulder; often above the umbilicus, differing from that of appendicitis. Subsequent attacks may be from the same stone. The direction of growth of the tumor is diagnostic, being obliquely toward the umbilicus. It may be enormous, sometimes, then, being mistaken for ovarian tumor. Distention of the gall-bladder without jaundice, means, obstruction of the cystic duct, as by enlarged gland, stricture, or stone. Sometimes a diagnosis may be made by the presence of stone in the fœces. Hemorrhage, when present, is due to poisoning incident to cholœmia. In this latter we have continuous jaundice, which means neoplasia of the duct, or head of the pancreas. Stone, by its change of position, etc., will allow the escape of some bile, jaundice accompanying it being thus intermittent.

The question of peritonitis is important. A stone near the papilla is a foreign body, and, as such, it irritates, and leads to the establishment of peritonitis, which may become purulent. Chills, fever, sweats, etc., occur here. Inflammation may result in perforation of the gall tract, setting up peritonitis thus. From malaria, differentiation is sometimes impossible. Dr. Taylor said he could recall cases in his own practice in which diagnosis was only made by the autopsy.

Solvents are myths, and the only medical indication is to relieve pain.

Dr. J. S. Wellford reported two cases of cholelithiasis. Most cases, said he, of gout commence with violent pain, especially in the right hypochondriac region, and this should be borne in mind in diagnosing gall-stone and appendicitis.

MARK W. PARKER, M. D.,
Secretary and Reporter.

